# Preliminary evaluation of image quality in a new clinical ToF-PET/MR scanner

**DOI:** 10.1186/2197-7364-1-S1-A41

**Published:** 2014-07-29

**Authors:** Gaspar Delso, Mehdi Khalighi, Marlena Hofbauer, Miguel Porto, Patrick Veit-Haibach, Gustav von Schulthess

**Affiliations:** GE Healthcare, Waukesha, USA; University Hospital, Zurich, Switzerland

A new ToF-PET/MR scanner has recently been deployed at the University Hospital of Zurich for clinical evaluation purposes. The goal of the present work was to compare the image quality obtained on this system with that of equivalent state-of-the-art standalone scanners.

The GE Signa PET/MR is a whole-body scanner combining a 3T wide-bore MR system with a 25 cm PET detector ring based on SiPM technology and mounted on a customized radiofrequency coil.

MR image quality of this system was tested by scanning two volunteers: One with a brain protocol and another with a whole-body protocol. The same scans were repeated before and after PET detectors were installed on the system. A baseline measurement was acquired on a Discovery 750w scanner with the same volunteers.

PET image quality was tested on patients referred for an FDG PET/CT scan. After 15-20 minutes of PET/CT, the patients were moved to the PET/MR, where an equivalent acquisition was performed.

**Figure 1:**
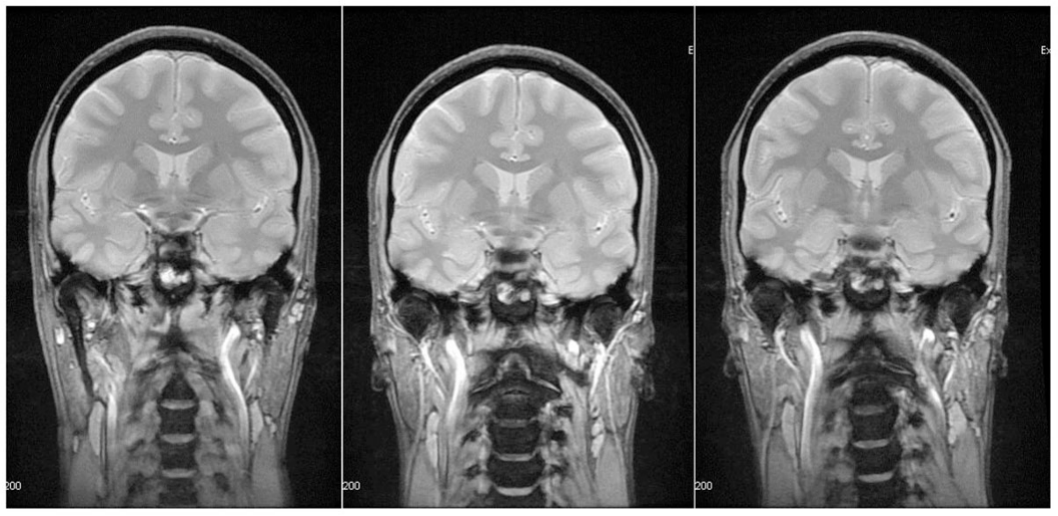
Coronal 2D MERGE views of a healthy volunteer scanned on a Discovery 750w (left), on the PET/MR before the installation of the PET insert (center) and on the same scanner once the insert was in place and online (right).

**Figure 2:**
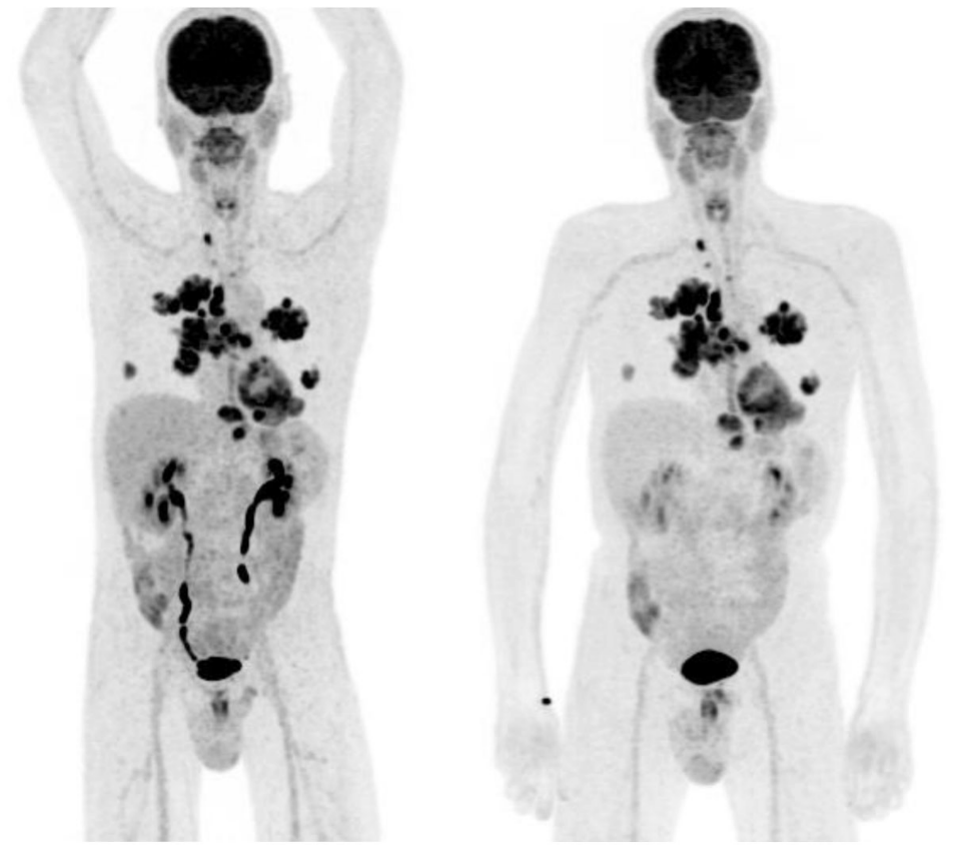
Three-dimensional maximum intensity projection of a PET/CT acquisition (left) and posterior PET/MR acquisition (right) of the same patient.

Data were reviewed by nuclear medicine and radiology physicians. As seen in Figure 1, MR image quality in the brain is identical between PET/MR (pre- and post-insert) and MR750w. In whole-body scans, shading on the images due to different receive coil sensitivity were observed. These are explained by the replacement of the bed-mounted posterior array coil by a central matrix array at the scanner iso-center. Figure 2 illustrates the differences in PET, showing equivalent or better quality in the PET/MR data, despite latter acquisition, justified by the increased sensitivity of the scanner.

Our results show that the image quality of clinical MR sequences is not affected by the presence of the PET detector insert. PET image quality shows great promise, but will require further testing to confirm this quantitatively.

